# A Comparative Study on the Effects of Au, ZnO and AZO Seed Layers on the Performance of ZnO Nanowire-Based Piezoelectric Nanogenerators

**DOI:** 10.3390/ma12162511

**Published:** 2019-08-07

**Authors:** Camille Justeau, Taoufik Slimani Tlemcani, Guylaine Poulin-Vittrant, Kevin Nadaud, Daniel Alquier

**Affiliations:** GREMAN UMR 7347, Université de Tours, CNRS, INSA Centre Val de Loire, 37071 Tours, France

**Keywords:** seed layer, ZnO nanowires, piezoelectric nanogenerator, hydrothermal method, harvesting energy

## Abstract

In this study, different seed layers like gold (Au), zinc oxide (ZnO) and aluminum-doped ZnO (AZO) have been associated to ZnO nanowires (NWs) for the development of mechanical energy harvesters. ZnO NWs were grown by using a low temperature hydrothermal method. The morphological properties were investigated using Scanning Electron Microscopy (SEM) and the analysis of crystalline quality and growth orientation was studied using X-ray Diffraction (XRD). The obtained ZnO NWs are found to be highly dense, uniformly distributed and vertically well aligned on the ZnO and AZO seed layers, while ZnO NWs grown on Au possess a low density and follow a non-uniform distribution. Moreover, the NWs exhibited good crystal quality over the seed layers. The piezoelectric nanogenerator (PENG) consists of ZnO NWs grown on the three different seed layers, parylene-C matrix, Ti/Al top electrode and poly(dimethylsiloxane) (PDMS) encapsulated polymer composite. The measurements of the open circuit voltage (V_OC_) were around 272 mV, 36 mV for ZnO, AZO seed layers while the PENG including Au seed layer presented a short-circuited state. This study is an important step in order to investigate the effect of different seed layers influencing the magnitude of the generated electrical performances under identical growth and measurement conditions. It will also help identify the most suitable seed layers for energy harvesting devices and their future integration in industrial applications.

## 1. Introduction

Owing to the fast increasing energy concerns and the extensive use of energy resources at large scales, there is a growing attention toward the development of micro energy (environment friendly) storage and conversion systems. In particular, the development of energy conversion systems such as nanogenerators have aroused a great challenge of research interest to overcome the high demand for sustainable micro energy sources, with the rise of the Internet of Things [1,2].

From the literature reviews, it is obvious that the vibration sources obtainable in our daily life, for instance, human body movements, wind gradient, water flow, vehicle motion, etc. can be used as mechanical source to generate energy, owing to its availability [3,4,5,6]. Furthermore, this mechanical energy is ubiquitous in the environment and more accessible than other renewable energy resources, such as thermal and solar [7,8,9]. Several types of nanogenerators like piezoelectric [7], triboelectric [5], thermoelectric [10] and pyroelectric ones [11] are reported in the literature. Among these, the piezoelectric nanogenerators (PENGs) have attracted much attention due to their unique working principle [12], which is independent of external environmental conditions such as temperature and humidity [7]. When the electrical load supplied by the PENG operates intermittently, the PENG can be hybridized with small energy storage systems, for example micro-batteries or super-capacitors [13]. 

In recent times, different materials have been studied in order to fabricate optimized nanogenerators for harvesting a sufficient amount of mechanical energy [12,14,15]. The piezoelectric effect can be generally detected in the inorganic materials, for instance barium titanate (BTO), lead zirconium titanate (PZT), zinc oxide (ZnO), etc. [15,16,17,18,19,20,21]. Among these inorganic piezoelectric materials, the PZT has the highest piezoelectric constant but it contains of lead which has toxic effects on human health and environment, which encourages to study lead free piezoelectric materials [22]. ZnO is emerging as an attractive material for a wide range of applications, as it exhibits both piezoelectric and semiconducting properties [23,24]. In addition, ZnO has a bio-safe nature, presents diverse and abundant configurations of nanostructures [25] and can be synthesized using simple techniques at low temperature. Thus, various reports have been published on the piezoelectric properties of ZnO nanostructures, such as nanobelts (NBs), nanoflowers (NFs), nanorods (NRs) and nanowires (NWs) [26,27,28,29,30,31]. Until now, piezoelectric ZnO NWs have been the most outstanding candidate for nanogenerators due to their one dimensional (1D) structure which has a high sensitivity to small force and large deformation and can also provide a significant enhancement in the mechanical flexibility and optical transparency. Moreover, by optimizing the alignment and the density of the NWs on the seed layer, a piezoelectric nanogenerator performance could be greatly enhanced [32]. This study illuminates the use of different seed layers in the architecture of ZnO NWs based PENG. Although the effect of seed layer on the growth properties of ZnO NWs has been reported in the literature [31], the influence of different seed layers on the performance of ZnO NWs based PENG has not been addressed yet. Such investigation has the great potential to provide valuable information on the fabrication and the characterization of nanogenerators in order to improve their performances and hence to tailor the development of the related PENGs. Our devices are aiming at harvesting low amplitude and low frequency mechanical sources such as human movements or forces, or fluid flows, for example in medical applications (pacemaker), intelligent buildings (smart lockers), or environmental health monitoring (wind or water).

In this paper, the ZnO NWs are grown using a hydrothermal method, which is most advantageous owing to its simplicity, low cost, convenience, and growth at relatively lower temperatures [31,33]. Herein, we report the PENGs fabricated using ZnO NWs. A large amount of high-quality ZnO NWs can be produced after one reaction at low temperature and the NWs were grown homogeneously over the entire seed layers with a hexagonal wurtzite structure and high density along the c-axis direction. Furthermore, in this study, gold (Au), ZnO, and aluminum-doped ZnO (AZO) seed layers have all been used for the growth of ZnO NWs in order to analyze the effect of seed layer on the magnitude of the output voltage supplied by the PENG when submitted to an external force. We aim to better understand and enhance the piezoelectric potential and consequently to optimize future PENG prototypes. Details of the growth of ZnO NWs and the characteristics of the PENGs are also discussed.

## 2. Materials and Methods

### 2.1. Seed Layers Preparation

The study was performed with three samples gathered in Table 1 and named A, B, C respectively for Au, ZnO, and AZO seed layers. Regarding the size, 2 × 2 cm^2^ samples were used. Information regarding reagents and equipment used can be found in Appendix A.

Seed layers A and B were deposited on n-type, 500 µm thick silicon (Si) wafer (100) cleaned in a mixture of sulfuric acid and hydrogen peroxide bath (H_2_SO_4_:H_2_O_2_, 1:1) for 10 min at 110 °C followed by 2 min in diluted hydrofluoric acid HF (25%) to remove any traces of metallic and organic contaminants as well as the native oxide SiO_2_ layer [34]. A 100 nm thick adhesive titanium (Ti) layer was DC sputtered on sample A by magnetron plasma at 500 Watts, under 5 mTorr pressure in argon atmosphere in order to improve interfacial bonding between the Si (100) and Au (111) layers [35]. Then, the 200 nm Au layer was added following the same protocol to serve both as seed layer for ZnO NWs synthesis and bottom electrode for the PENG applications. 

Sample B followed the same steps for metallic deposition to keep the Ti/Au layer serving as bottom electrode and additionally a 100 nm thick ZnO seed layer was radio-frequency sputtered on the gold surface (65 W, 5 mTorr) under argon atmosphere.

Regarding seed layer C, commercial AZO coated glass was cleaned for 8 min in an ultrasonic bath of isopropanol (IPA) before being used as is for both bottom electrode and seed layer purposes. The interest behind the use of commercial AZO layer is to fabricate a PENG on a seed layer already industrially optimized to better understand how the AZO layer could replace at once the bottom electrode and the seed layer for the hydrothermal growth of ZnO NWs.

### 2.2. ZnO Nanowires Synthesis

**Figure 1:**
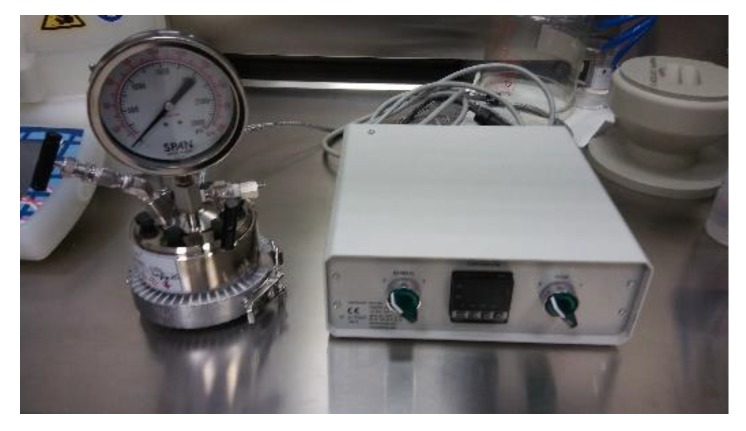
Hydrothermal growth set up with the stainless steel autoclave and temperature regulator.

The entire procedure for the hydrothermal synthesis of ZnO NWs on the substrates was reported in detail by our team in a previous article by Tlemcani et al. [31]. The growth was carried out in a temperature controllable autoclave (Figure 1) in which two fresh and equimolar stock solutions of Zn(NO_3_)_2_ and HMTA were mixed to reach a solution concentration of 34 mM, including a 0.8 mM addition of ammonia solution. The pH of the obtained transparent growth solution was known to be around 7.4. 

After placing the substrates tilted against the walls of the autoclave to prevent the precipitation of homogenous nucleated ZnO on the active surface, the reactor was hermetically sealed and the system was heated up for 30 min, to reach the targeted temperature of 85 °C, and was held for 3.5 h. The real temperature ensured inside the growth solution was actually about 70 °C. After growth, the samples were taken out, rinsed by spraying deionized (DI) water and dried under air flow.

### 2.3. Nanogenerator Fabrication

**Figure 2:**
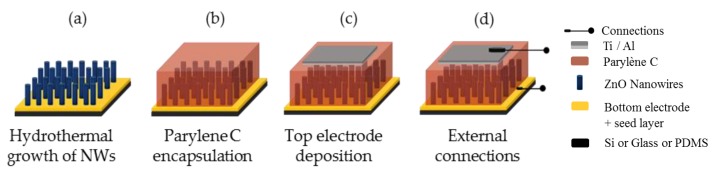
Piezoelectric nanogenerator (PENG) fabrication process (**a**) hydrothermal synthesis of ZnO NWs; (**b**) parylene C encapsulation; (**c**) top electrode deposition and (**d**) connection of the wires.

The PENG fabrication process was described in Figure 2 starting from the substrate on which ZnO NWs were grown by hydrothermal synthesis (Figure 2a). The first step consisted of wrapping the NWs into a parylene C polymer matrix that would ensure the mechanical integrity and efficiency of the device (Figure 2b). Indeed, this polymer matrix was a key point in the development of an electrically efficient PENG for two main reasons. The primary role of the parylene C, mainly due to its low Young Modulus (2.8 GPa) [36], was to correctly infiltrate into the sample to insulate the NWs from each other and reduce the impact of screening effects on the piezoelectric properties of the ZnO NWs [37,38]. The secondary role fulfilled by the polymer was to create a capacitive coupling between the NWs and the top electrode. In fact, during the deposition process, a thin layer of parylene C was also covering the top surface of the NWs to both help in improving the conformal cover at the interface [39] and to create a capacitive coupling. The thickness of the parylene layer above the NWs needed to be very thin without losing its insulating properties. The parylene C was deposited according to the Gorham procedure [40] known now as Vacuum Deposition and Polymerization Process (VDP). 

In a second step, a top electrode was deposited at the surface of the parylene C. To do so, the surface of the samples was masked to define the area of the top electrode around 1.2 cm^2^, which was thus the active area of the PENG. Then, a treatment of the parylene uncovered surface was done in O_2_ plasma for 5 s to improve the bonding properties of the surface to the metal electrode [41,42]. Finally, a 100 nm thick adhesive titanium layer was deposited by evaporation, before adding a 400 nm aluminum layer following the same protocol (Figure 2c).

In order to connect the PENG to an external load as well as to assess its electrical performances, conductive epoxy silver paste was used as a fourth step to attach copper wires to the bottom and the top electrodes of the PENG (Figure 2d). 

Before completing the last fabrication step, the samples were put in the oven at 90 °C for one hour to remove any trace of moisture. Then, a final encapsulation of the device was made by spin coating 400 mg of degassed PDMS at 500 rpm for 15 s on the sample surface and curing it on a hot plate at 100 °C for 45 min before putting it in the oven at 90 °C overnight.

**Figure 3:**
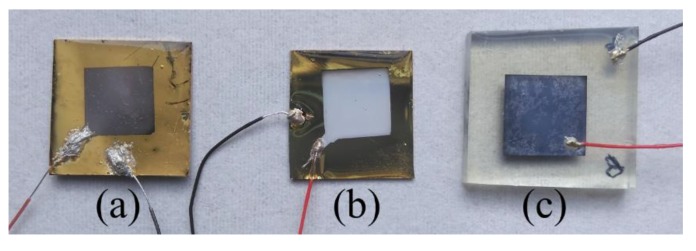
Images of PENGs made from ZnO NWs grown on (**a**) Au; (**b**) ZnO and (**c**) AZO seed layers.

This encapsulation was performed to both improve the durability of the PENGs during the electrical characterizations and protect them from external factors. The visual aspect of the PENGs made from the ZnO NWs grown on different seed layers are shown in Figure 3.

### 2.4. Characterizations

Structural characterizations of the Au, ZnO and AZO seed layers as well as ZnO NWs were performed using an X-ray Diffraction (XRD) with a CuK1 radiation on the high-resolution parallel beam diffractometer Brucker AXS D8 discover (Bruker, Karlsruhe, Germary). The morphological characterizations of the ZnO NWs surface and cross section were done by scanning electron microscopy (SEM) with a JEOL JSM-7900F (HRSEM, Croissy-sur-Seine, France). The extraction of the densities, lengths and diameters associated with the ZnO NWs was obtained using Image J software on SEM images [31]. The surface topography of the seed layers was measured with a Dimension ICON Brucker Atomic Force Microscope (AFM, Bruker France SAS, Palaiseau, France). The resistivity of each seed layer was analyzed with a semi-automatic hall effect measurement system HMS 5000 Microworld (Microworld, Grenoble, France).

**Figure 4:**
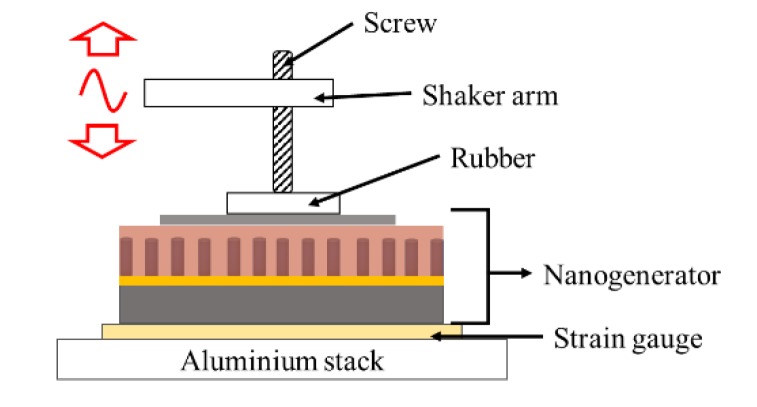
Measurement test bench for PENG performances.

The electrical performances were assessed using a test bench (Figure 4) designed by our team and described in detail by Oshman et al. [43]. The PENG was being solicited by the aluminum mechanical arm impacting its surface on 1 cm^2^ area and was connected to a double buffer circuit consisting of a differential amplifier that measured the output voltage without applying any parasitic bias [20].

## 3. Results

### 3.1. Structural Characterizations

**Figure 5:**
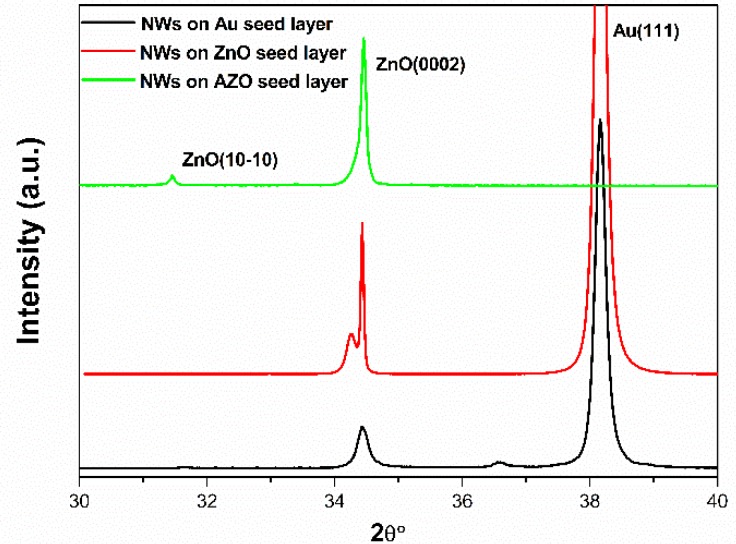
XRD patterns of the ZnO NWs grown on Au, ZnO and AZO seed layers before the parylene C coating step.

First of all, the XRD patterns of the ZnO NWs grown on Au, ZnO and AZO seed layers are shown in Figure 5. From these three spectra, we can clearly see that the XRD patterns of ZnO NWs grown on three different seed layers all clearly exhibited the strong diffraction peaks at 34.4° corresponding to the (0002) plane of ZnO. All the diffraction peaks could be indexed to the hexagonal wurtzite phase of ZnO NWs and the obtained results were in good agreement with the standard JCPDS file No. 05-0664. No impurity peak was found in the XRD patterns, which revealed that the ZnO NWs synthesized by our method were pure phase.

But in spite of that, Figure 5 revealed that the intensity of the (0002) peak varies with respect to the seed layer. Moreover, the XRD pattern of ZnO NWs grown on Au seed layer showed that the intensity of (0002) peak was relatively low compared with the other seed layers. With the use of the ZnO and AZO seed layers, the increased (0002) peak intensity and decreased full-width at half-maximum (FWHM) indicated an improvement in the crystallinity of the NWs. It is well known that in order to grasp the crystallization of ZnO NWs, the selection of the seed layers is an essential factor to be taken into account [44,45]. However, the XRD pattern of ZnO NWs grown on ZnO seed layer showed (0002) peak splitting, which could be reasonably due to the large lattice mismatch between NWs and seed layer. This was discussed in detail in our previous study [31]. Therefore, the highly intense (0002) peak of ZnO NWs grown on ZnO and AZO seed layers respectively is providing clear evidence that both these seed layers are more favorable for the growth of ZnO NWs. These results were consistent with the following scanning electron microscopy (SEM) observations.

**Figure 6:**
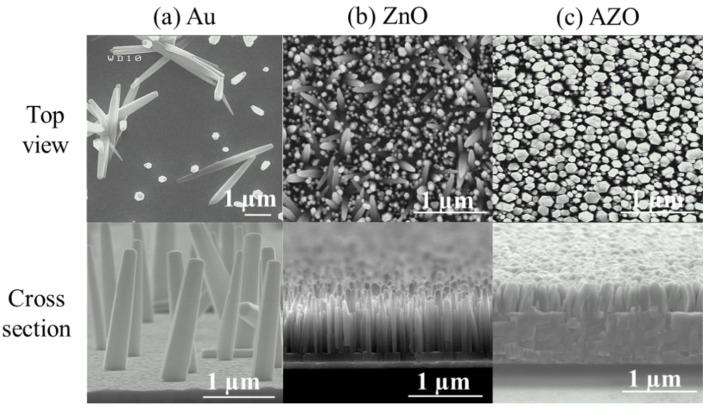
SEM images of ZnO NWs grown on (**a**) Au; (**b**) ZnO and (**c**) AZO seed layers.

Figure 6 shows the SEM images in top-view and cross-section of ZnO NWs grown on different seed layers including Au, ZnO and AZO, respectively. It is noted that the use of different seed layers led to a remarkable change in the morphology of the ZnO NWs. The images also show the good alignment of hexagonal faces ZnO NWs, with a high density and a perpendicular orientation to the substrate when adopting ZnO and AZO as a seed layer. The cross-sectional views of the samples show that almost all ZnO NWs (Figure 6b,c) are perpendicular to the seed layer. Figure 6a shows the randomly oriented ZnO NWs which were grown on Au seed layer. Therefore, ZnO and AZO seed layers had a strong effect on the aligned growth of ZnO NWs as evidenced by XRD pattern which revealed that NWs grown on Au seed layer had a lower crystallinity. It is well known that the integration of catalysts, such as Au, Cu and Ag, etc. on the ZnO seed layer improves the growth of ZnO NWs [46,47]. Therefore, the Al dopant could act as a catalyst for improving the growth quality of ZnO NWs. These interesting results enlighten us that well-aligned ZnO NWs can be formed not only on the ZnO seed layer but also on the AZO seed layer substrate.

The ZnO NWs on the Au, ZnO and AZO seed layers had an average density of 0.6, 35.4 and 52.9 NWs per μm^2^, respectively. The adoption of ZnO and AZO seed layers led to the increase of the ZnO NWs density with a decrease of length and diameter (Table 2) that could mainly be explained by mass transport phenomenon [48] and change of nucleation sites [46]. 

**Figure 7:**
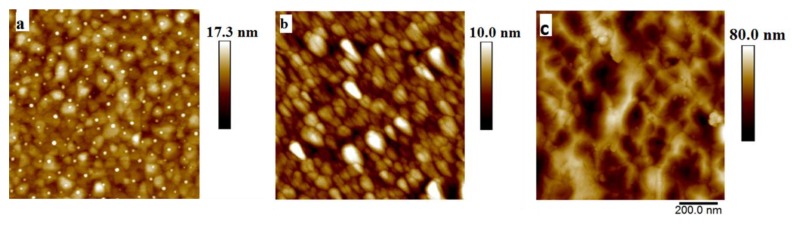
AFM images of three types of seed layers: (**a**) Au; (**b**) ZnO and (**c**) AZO.

Furthermore, the density and the alignment of the NWs could be improved with the larger crystal size of the seed layer as shown in the two-dimensional (2D) AFM images of the surfaces of different seed layers (Figure 7a–c). It was reported that the alignment and density are essential morphological characteristics of the NWs, and these characteristics have a crucial role in the properties of the NW-based devices [46,49,50].

### 3.2. Piezoelectric Energy Harvesting Device Performance

**Figure 8:**
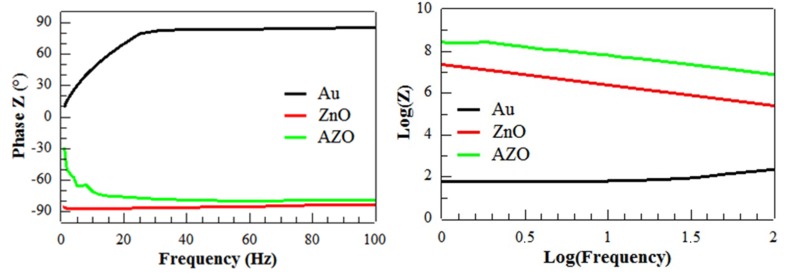
Curves extracted from impedance measurements showing (**a**) the log(Z) vs. log(frequency) and (**b**) the Z phase vs. the frequency for PENGs made from ZnO NWs grown on Au, ZnO and AZO seed layers.

The impedance measurements done on the PENGs built from ZnO NWs grown on Au, ZnO and AZO seed layers helped to determine the internal impedance of the PENG as well as to detect any short-circuit. To do so, an AC voltage of 200 mV_pp_ was applied to the PENG and the AC current flowing through the PENG was measured over a frequency range from 1 Hz to 100 Hz. From the recorded data, the complex impedance Z was extracted for each device by calculating (i) the ratio between voltage and current, to obtain the modulus, and (ii) the phase shift between voltage and current, to obtain the argument. The curves presenting the plot of the logarithm of Z modulus depending on the logarithm of the applied frequency are given in Figure 8a. These curves indicate the type of internal impedance of the PENG device (capacitive, inductive and/or resistive) and allow to detect if the PENG device is short-circuited. The impedance slope values that tends toward −1 (−0.98 and −0.81 respectively) visible on Figure 8a proves a fully capacitive response of the PENGs integrating the ZnO and AZO seed layers. However, the PENG built from Au seed layer clearly shows a resistive behavior corresponding to a short-circuited device.

The −90° value, reached for the Z phase in Figure 8b, confirms the capacitive response of PENGs integrating the ZnO and AZO seed layers. In addition, the Z phase value of almost +90° given by the PENG integrating Au seed layer demonstrates an inductive behavior and confirms the short-circuited state of the device.

**Figure 9:**
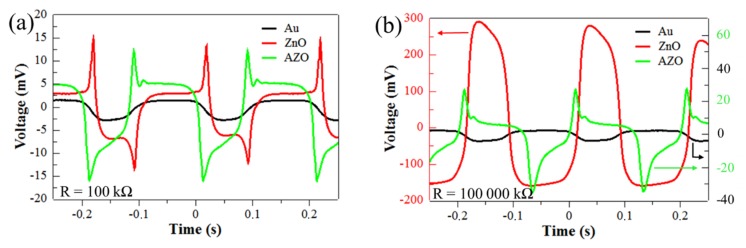
Output voltage response of PENGs based on Au, ZnO and AZO seed layers at a resistive load of (**a**) 100 kΩ and (**b**) 100,000 kΩ with a 3 N force applied at 5 Hz.

To mimic real energy harvesting conditions of micro energy sources and to determine their maximum available power, the PENGs were placed in the test bench (Figure 4) to impact the surface of the PENGs with a 3 N amplitude force at a frequency of 5 Hz. The PENG response was recorded for a resistive load range of 100 to 100,000 kΩ using an adjustable resistance. Figure 9 presents the three PENGs voltage outputs measured at 100 kΩ (Figure 9a) and 100,000 kΩ (Figure 9b) resistive load values. For device B (with ZnO seed layer) and device C (with AZO seed layer), the resistive load value greatly affected the PENGs output voltage amplitude: for device B, the positive peak amplitude was 15 mV and 292 mV at 100 kΩ ant 100,000 kΩ respectively, with also very different signal shapes. This underlines the influence of the resistive load value on the device voltage response: the device B (with ZnO seed layer) presented at 100,000 kΩ a voltage signal with a slow decrease, due to the fact that 100,000 kΩ wasmuch superior to the internal impedance of the device (Figure 8a), thus the PENG device was in open-circuit condition; the device C (with AZO seed layer)was not in open circuit as its internal impedance was higher than the 100,000 kΩ load, thus the voltage signal presented a fast decrease after each peak. Moreover, devices integrating ZnO and AZO seed layers clearly exhibited a voltage response to the mechanical solicitation that is typical of piezoelectric devices while the device integrating Au seed layer did not: when the resistive load value changed, its response was not significantly impacted (with positive and negative voltage peaks below 4 mV), which confirmed the short-circuit state observed during the impedance measurements. The recorded output signal was due to the noise in the measurement set-up.

In addition, the PENGs electrical output characteristics, such as open-circuit voltage (V_OC_), short-circuit current (I_SC_), peak power (P_pk_) and optimal load resistance (R_opt_), were gathered in Table 3 and confirmed the higher performances of the PENG including ZnO seed layer compared to the one including an AZO seed layer (V_OC_ value 272 mV and 36 mV respectively). 

**Figure 10:**
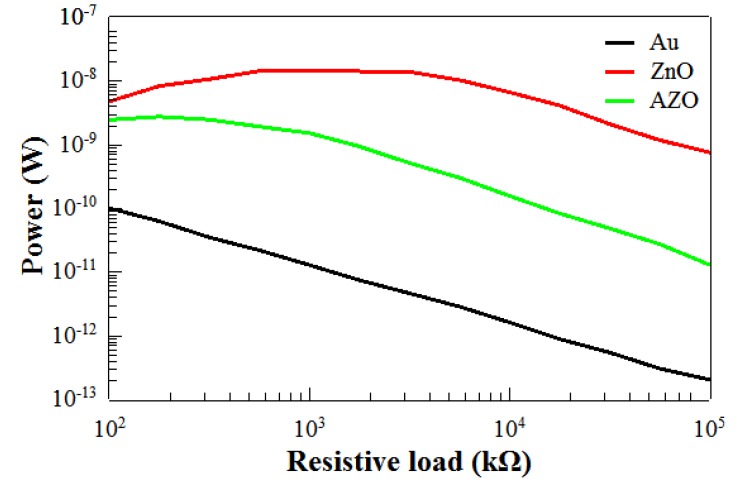
Peak power generated by PENGs including different seed layers. Power measured for a resistive load ranging from 100 to 100,000 kΩ.

The output peak power curves given in Figure 10 show a maximum of power given for an optimal value of resistance for the PENGs based on ZnO and AZO seed layers. This underlines the shape expected from piezoelectric PENGs: an optimum load value, corresponding to the maximum power, was observed around 177 kΩ for sample C, around 562 kΩ for sample B. This did not correspond to the values of Z modulus shown in Figure 8a. This may be explained by the fact that the applied force was not sinusoidal, and the contribution of harmonics modified the frequency response of the PENG as demonstrated by Nadaud et al. [51]. Moreover, the output peak power curve of the PENG based on Au seed layer did not give any maximum and was localized in the noise level of the measurement set up. This confirms the short-circuit state of the device, which could be explained by the poor density and alignment of the grown ZnO NWs, reported in Table 2 and described through the SEM images in Figure 6. In fact, this lack of uniformity in the density and morphology of the ZnO NWs does not ensure a good conformal interface between the bottom and top electrodes of the device [52]. The lack of alignment of ZnO NWs could cause difficulties regarding the conformal deposition of parylene C. In fact, with ZnO NWs being naturally n-doped and thus quite conductive, the areas where they are not well covered by the insulating polymer may create intrinsic connections between the electrodes.

Regarding the AZO seed layer leading to the best morphology and density compared to ZnO and Au seed layers, the resulting PENG piezoelectric potential was expected to be the highest of the three PENGs. Consequently, the best electrical performances were expected for the AZO device. The surprising poor performances might be explained by several factors. A first hypothesis could be that the conductivity of AZO seed layer was too low due to its small thickness, preventing it from being a functional bottom electrode. AZO may present a series resistance which is too high compared to Au, and this could result in bad electrical charges transfer and increase the electrical losses. However, when measuring the resistivity of AZO and Au by Hall effect, the respective values were found to be 3.841 × 10^−4^ and 3.078 × 10^−6^ Ω·cm, which demonstrate that the AZO exhibits a remarkable potential to act as an electrode. Another interpretation can come from the morphology of ZnO NWs. In fact, when comparing the aspect ratio, defined by the ratio of length over diameter and shown in Table 2, the NWs grown on AZO exhibited the lowest value with 3.4, compared to a ratio of 8 for NWs grown on ZnO seed layer. This shows that the better uniformity and density obtained for NWs grown on AZO has also led to a decrease of their aspect ratio, maybe inducing a higher rigidity of the NWs array. As mentioned in the literature [53], high aspect ratio is needed to allow large deformation of the NWs and to get efficient electro-mechanical energy conversion. The AZO seed layer clearly shows good potential to replace both the Ti/Au electrode and ZnO seed layer, however some adjustments still must be done in order to fulfill the two objectives: a seed layer acting as a good quality bottom electrode and high aspect ratio ZnO NWs.

## 4. Conclusions

In summary, ZnO NWs were grown using hydrothermal synthesis, on Au, ZnO and AZO seed layers and integrated to functional piezoelectric PENGs. The composition and characteristics of the seed layers play a significant role in the growth and structure of ZnO NWs especially regarding their alignment, density and aspect ratio. ZnO and AZO seed layers were found to give higher homogeneity to the NWs growth. The evaluation of electrical output performances of the subsequent PENGs was done using a homemade test bench and clearly confirmed better results of PENGs including ZnO seed layer, with an output voltage peak value of 292 mV at a resistive load of 100,000 kΩ. Despite lower electrical performances due to low aspect ratio of the NWs, electrical characterization of AZO seed layer underlined their good potential for integration into PENGs as a substitute for both bottom electrode and seed layer. With future adjustments on NWs aspect ratio, such findings are expected to offer great benefits for industrial applications. In fact, the replacement of metallic electrode and ZnO seed layer by AZO would provide a time and cost saving solution that would also limit the use of metallic resources. Finally, using AZO as a bottom electrode gives good perspectives regarding the adaptability of the process toward flexible piezoelectric nanogenerators in order to avoid the potential delamination of metallic electrodes during bending operation for mechanical energy harvesting application. To go further, cycling tests can pave the way to evaluate the lifetime and robustness of the PENGs based on these different seed layers.

## Figures and Tables

**Table 1:** Overview of the seed layers under study and their thicknesses.

Sample	Seed Layer	Thickness (nm)	Substrate
A	Au	200	Si
B	ZnO	100	Si
C	AZO	500	Glass

**Table 2:** Characteristics of ZnO NWs grown on different seed layers.

Sample	Density (NWs/µm^2^)	Diameter (µm)	Length (µm)	Aspect RATIO
A (Au)	0.6 ± 0.1	0.38 ± 0.13	1.78 ± 0.20	4.7
B (ZnO)	35.4 ± 0.2	0.07 ± 0.03	0.57 ± 0.18	8
C (AZO)	52.9 ± 0.5	0.14 ± 0.06	0.48 ± 0.08	3.4

**Table 3:** Electrical performances of the PENGs depending on their seed layer.

Sample	V_OC_ (mV)	I_SC_ (nA)	P_pk_ (nW)	R_Opt_ (kΩ)
A (Au)	<4	<15	-	-
B (ZnO)	272	214	17	562
C (AZO)	36	157	3	177

## Appendix A

Hydrofluoric acid HF (50%), hydrogen peroxide H_2_O_2_ (30%), sulfuric acid H_2_SO_4_ (96%) and isopropanol were supplied by KMG ultrapure Chemicals SAS (Saint Fromond, France) and Technic France all of which used without further purification. The commercial AZO glass coated substrates were purchased from MSE Supplies (Tucson, AZ, USA). The polymer and curing agent needed to fabricate PDMS were obtained from Samaro SAS (Thouare, France). The chemicals used for the ZnO NWs growth included zinc nitrate hexahydrate Zn(NO_3_)_2_·6H_2_O (98%), hexamethylenetetramine (HMTA) (CH_2_)_6_N_4_ (>99.5%) purchased from Sigma-Aldrich S.a.r.l (Saint-Quentin Fallavier, France) and ammonium hydroxide NH_4_OH (29%) solution from KMG ultrapure Chemicals SAS (Saint Fromond, France) also used as received. The deposition of bottom metallic layers was done in a MP 650S Plassys physical vapor deposition (PVD) equipment, the top metallic layers in a MEB 550S Plassys PVD equipment and the ZnO seed layer deposition with a Plassys MP 550 S PVD equipment (Marolles-en-Hurepoix, France). A stainless steel temperature controllable autoclave from Parr Instrument Company (Moline, IL, USA) was used to operate the synthesis of ZnO NWs. The parylene C used for encapsulation was deposited in a SCS equipment from KISCO Company (Pliezhausen, Germany). The O_2_ plasma treatment was realized in a PVA TePla Microwave Plasma-systems 400 (Corona, CA, USA). The bi-component epoxy silver paste was supplied by FT Polymer SAS (Orgeval, France).
